# Organizational Barriers to Oral Health Conversations Between Health Visitors and Parents of Children Aged 9–12 Months Old

**DOI:** 10.3389/fpubh.2021.578168

**Published:** 2021-02-23

**Authors:** Ieva Eskytė, Kara A. Gray-Burrows, Jenny Owen, Bianca Sykes-Muskett, Sue H. Pavitt, Robert West, Zoe Marshman, Peter F. Day

**Affiliations:** ^1^School of Law, University of Leeds, Leeds, United Kingdom; ^2^School of Dentistry, University of Leeds, Leeds, United Kingdom; ^3^Occupational Health Department, University of Huddersfield, Huddersfield, United Kingdom; ^4^Dental Translational and Clinical Research Unit, School of Dentistry, University of Leeds, Leeds, United Kingdom; ^5^Leeds Institute of Health Sciences, University of Leeds, Leeds, United Kingdom; ^6^School of Clinical Dentistry, University of Sheffield, Sheffield, United Kingdom; ^7^Bradford Community Dental Service, Bradford District Care NHS Foundation Trust, Bradford, United Kingdom

**Keywords:** oral health, health visitors, parents, young children, barriers

## Abstract

**Background:** Dental caries is the most prevalent preventable childhood disease and a major public health priority. Local authorities in England have a statutory responsibility to improve child health, including oral health, through the “Healthy Child Programme.” The “Healthy Child Programme,” which includes the provision of oral health advice is delivered by health visitors to parents of young children. To date, research has mainly concentrated on individual interactions between health visitors and parents, with less attention given to the broader context in which these oral health conversations between health visitor and parents take place.

**Objective:** Our study explored the organizational factors that obstruct health visitors from engaging in meaningful conversations with parents about young children's oral health.

**Methods:** Qualitative interviews and focus groups were held with health visiting teams (*n* = 18) conducting home visits with parents of 9–12-month olds in a deprived, urban area in England.

**Results:** The study revealed the wide variation in what and how oral health advice is delivered to parents at home visits. Several barriers were identified and grouped into four key themes: (1) Priority of topics discussed in the home visits; (2) Finance cuts and limited resources; (3) Oral health knowledge and skills; and (4) Collaborative working with other professionals. It was evident that organizational factors in current public health policy and service provision play an important role in shaping oral health practices and opportunities for behavior change.

**Conclusion:** Organizational practices and procedures play an important role in creating interaction patterns between health visiting teams and parents of young children. They often limit effective engagement with and positive change in oral health. For future oral health interventions to be effective, awareness of these barriers is essential alongside them being founded on evidence-based advice and underpinned by appropriate theory.

## Introduction

Dental caries (tooth decay) is the most prevalent preventable childhood oral disease and a major public health priority (1, 2). Approximately a quarter of children aged 5 years old in England are affected (23.4%) with this figure rising to around half of children aged 8 years old (46%) (3). There are significant variations seen in the prevalence and severity of disease. For example in 5 year olds, the prevalence of dental decay was 13.7% in the least deprived areas compared to 34.3% in the most deprived areas (4). Caries causes pain and discomfort (5), dietary changes (6, 7), problems with speech development (8), and negatively impacts overall health (9), quality of life, self-esteem and social confidence (10, 11), as well as school readiness, attendance and educational outcomes (12). Caries is the most common reason for dental care under general anesthetic in young children, representing over 30,000 hospital admissions every year (13). This alone costs the NHS approximately £36 million a year (14), and the substantial cost of managing dental caries in children accounts for a significant proportion of the £3.4 billion annual spend on NHS dentistry (15).

Parents' attitudes and beliefs (16, 17), education (18), socio-economic status (19–21), culture (22), and family functioning (23) and composition (24, 25) are key determinants of children's oral health and oral health-related behaviors. However, the health and social care systems families interact with also have an influence over everyday oral health behaviors (26); and indeed, have a responsibility to educate and advise them on good oral health practices for the whole family (26). For instance, since 2012, local authorities have had a statutory responsibility to improve children's health, including oral health (27). Indeed, reducing caries prevalence in 5 year-olds is included as a key priority in the Public Health Outcomes Framework (1). Recent National Institute for Health and Care Excellence (NICE) (28) and Public Health England (PHE) (14) guidance to local authorities has recommended early-life interventions to prevent caries that are delivered by multiple early-years professionals. One of the opportunities via which parents may engage with oral health advice is through their health visitors (29, 30).

In England, health visitors work mainly with parents of young children (0–5 years old). They provide a universal home visiting service that focuses on prevention, early intervention and parenting support. These early interventions for the family have been developed through the “Healthy Child Programme” (HCP) (31) and are divided into two levels: (1) “Community” and “Universal”; which are aimed at all families and includes community capacity building; and (2) “Universal plus” and “Universal partnership plus”: aimed at enhanced service provision for families who have additional needs (32). In this way all families are provided with a universal service, and some receive supplementary services according to their needs. Specifically, the Universal Plus delivers a “rapid response from the health visiting team when specific expert help is needed, e.g., with parental mental health, attachment, toilet training, behavior management, domestic abuse” (33). Universal Partnership Plus provides ongoing support from the health visiting team who works together with a range of local services and organizations to support families who have complex additional needs (33).

A universal home visiting service, includes support provided on topics such as transition to parenthood and the early weeks with your baby; maternal and paternal mental health; breastfeeding; healthy weight; managing minor illnesses and reducing accidents; health, well-being and development of a child aged 2 years old; and support to be “ready for school” (34). Oral health is a part of these conversations (34), especially with the eruption of primary teeth from around 6 months old. The 9–12 month home visit provides health visitors with the opportunity to communicate key oral health messages in accordance with the PHE and NICE guidance (28, 35). The guidance focuses on dental attendance, toothbrushing with fluoride toothpaste, healthy eating and reduction of dietary sugar (36). In some local authorities in England these conversations include the provision of fluoride toothpaste and toothbrush packs.

Even though health visitors are expected to provide parents with oral health knowledge and foster skills to improve home-based oral health behaviors, there is limited evidence of how effective these oral health conversations are (15, 27). Lewney et al. (37) and Oge et al. (38) suggest that some health visitors have never received any formal training about oral health. Other studies provide some evidence that health visitors may lack knowledge on children's oral health and this has a negative impact on their professional confidence (29). While they acknowledge that oral health should receive more attention, commitments such as child protection and immunization are often prioritized given the limited time for visits (37, 39). Coll et al. (29) found the oral health advice provided is often basic and reactive as opposed to comprehensive and proactive.

Health visitors can therefore play an important role in communicating oral health messages clearly and consistently in order to initiate and maintain good home-based oral health behaviors and thus reduce the level of tooth decay (30, 40). Our research (41–44) following complex intervention methodology, has led to the development of a logic model and generic oral health intervention underpinned by psychological theory (41). This work has identified individual, interpersonal, contextual, organizational, and community barriers to the initiation and adoption of appropriate oral health behaviors by parents of young children. Implementation research and methods focus on these wider barriers as these strongly impact on the successful implementation of new initiative within organizations (45, 46). The importance of organizational barriers has received less attention despite the critical role they play, especially in the context of health care service provision. In fact, there is a paucity of research that goes beyond issues, such as how parents' awareness and commitment to toothbrushing impact children's oral health or how accurate health visitors' knowledge is about young children's oral health. For instance, what do we know about how factors, such as organizational practices and procedures, that often do not depend either on health visitors or parents, shape home visits and engagement in oral health conversations? The aim of this article is therefore to explore how certain organizational practices affect interaction between health visitors and parents and shape their conversations about young children's oral health.

## Methods

This article reports data from a qualitative study that combined focus groups (*n* = 3; focus group 1—five professionals, focus group 2—four professionals; focus group 3—six professionals) and semi-structured interviews (*n* = 3) with health visiting teams working in a deprived urban area in England. The qualitative data of this participant group is used to explore the impact of organizational factors on oral health conversations between health visiting teams and parents of young children. It enables demonstration that the organizational framework within which these conversations take place is an important factor shaping not only the quality of the interaction but also the final outcome—young children's good oral health.

### Sample

The sample comprised of members of health visiting teams, including health visitors and nursery nurses who deliver universal home visits to parents of young children aged 9–12 months. It is important to acknowledge that nursery nurses are increasingly being utilized to deliver health visiting services, although this is dependent on geographical location and funding. Nevertheless, it was deemed prudent to include all individuals within the health visiting workforce to gain a comprehensive idea of the barriers faced when delivering oral health advice. Recruitment occurred in a deprived urban area in West Yorkshire where the prevalence of childhood dental caries is amongst the highest in England (31). Potential participants were approached via email or phone by the Health Visiting Lead of the project. The initial response was positive, with only two health visitors and one nursery nurse declining to participate on the day of the interviews/ focus groups.

### Procedure

An interview guide was devised by the authors based on previously published work (41–44) and aimed to explore health visitors and nursery nurses' practices and experiences when delivering oral health advice. Semi-structured questions were used to guide the discussion. Participants were invited to share their experiences and perspectives regarding oral health advice provision, as well as the challenges and opportunities in this process. Face and content validity of the guide were established, and the structure and questions were peer-reviewed by experts from disciplines including pediatric dentistry, dental therapy, social policy and behavioral psychology.

The focus groups and interviews were facilitated by the project manager (IE), assisted by a dental therapist (JO) and behavioral psychologist (BS-M). They took place in locations accessible and convenient to the participants, such as community centers, NHS Foundation Trust offices and other public settings that ensured confidentiality of the conversations.

The decision to utilize both focus groups and interviews to collect the data was two-fold. First, the use of focus groups allowed a broad approach to be taken, but more focused and in-depth discussion was provided by interviews. Second, on a practical level it was not always possible to gather all the health visiting teams together, therefore this approach was taken to allow everyone to have an opportunity to have their say on the subject.

At the end of the interview/focus group, participants were encouraged to ask questions about toothbrushing, healthy eating, oral health, and dental attendance. This established more equal power relations between the researchers and the participants, as the flow of information was bi-directional, with both parties acting as information providers. This shifted power between the researchers and the health visitors and nursery nurses creating a more equal interaction. This also served to provide better insight into the participants' knowledge about children's oral health, as by asking the researchers oral health questions it revealed more details on the depth of the participants' oral health knowledge. For example, some of the participants said that the guidance they follow says to use a pea-sized amount of toothpaste for children 3+ years old. However, during the discussion, it became apparent that their views on the size of a pea differed greatly. Data collection continued until no new themes emerged and data saturation was achieved. The discussions lasted between 50 and 75 min and were audio-recorded for subsequent transcription.

### Ethical Considerations

Ethical approval for the study was obtained from the University of Leeds Dental Research Ethics Committee (DREC: 160517/PD/229). All data were subject to procedures to ensure anonymity and confidentiality.

### Data Analysis

An inductive approach was taken to coding transcripts, combining manual coding with the use of the computer software “QSR NVivo 10.” To explore the range of issues identified during the interviews and focus group discussions, a thematic approach was adopted and the analysis of health visitors' accounts was primarily related to organizational barriers. This process involved careful reading and re-reading (47) of the transcribed data, aiming to identify the main themes. As well as identifying patterns within the data (48), individual or unique cases were noted down. The interviews were repeatedly read, aiming to find commonalities or contradictions among these unique cases (49, 50). Emerging themes were discussed within the research team, promoting rigor and facilitating consensus on the appropriate categories and sub-categories (49–51).

## Results

The workforce participants included health visitors and nursery nurses working within a deprived urban area in West Yorkshire; a population with high ethnic diversity (https://www.gov.uk/government/statistics/local-authority-health-profiles-2019). The participants (*n* = 18) ranged in ethnicity, age and experience (e.g., from a qualifying student to a retiree). All but one had received some oral health training, with at least one colleague receiving more in-depth training as this was of specific interest to her.

Similar and contradicting views were shared regarding the oral health advice provision at the 9–12-month reviews. While some discussions centered on the role of parents/carers in ensuring good oral health for children, a great deal of attention was dedicated to practices shaped by public health policies and organizational procedures in line with the focus of the present paper. While this reflects the complexity of the practice, there were pivotal themes, which resonated with all respondents. Four key themes were identified. These were: (1) Priority of topics discussed in the home visits; (2) Finance cuts and limited resources; (3) Oral health knowledge and skills; and (4) Collaborative working with other professionals.

### Priority of Topics Discussed in the Home Visits

All participants reported that they address child oral health at the 9–12-month universal home visits. While many of them noted that oral health is important, it was evident that when compared to other topics discussed at the developmental reviews, its importance was low. Participants' narratives suggested that current requirements for the visits, and parents' needs and preferences are key-factors shaping the practice.

Regarding current requirements in the area from which the participants were drawn The Personal Child Record, better known as the “Red Book,” was identified as a key-guide for the visits.

“*I would go through the development assessment in the red book first. And then I come on to dental health and then we give the book-start packs [a literacy initiative] out at the same visit as well.”*

The “Red Book” serves to guide the conversation. However, participants felt the content and level of focus on oral health in the “Red Book” had reduced over the years. The version available in West Yorkshire neither provides advice to parents on how to take care of their child's teeth, nor covers advice provided to parents by health care professionals. The outline of the 1 Year Review briefly notes that the child should now have their first tooth and be used to having their teeth brushed with a fluoride toothpaste. The guide does not, however, invite parents to discuss oral health with their health visitor, unlike other topics, such as the child's growth or weight; vision or hearing; sleep routines; behavior; and child development. Such an approach seems to be translated into the health visitors' practice:

“*Development, we do developmental review […]. Most of us start off with looking at are they meeting their milestones, gross and fine motor skills, starting to communicate. I usually talk about diet and what they're eating now, progressing with weaning. Covering dental health, home safety, do all the growth measurements. We do weight, head circumference and length. I pick up on maternal mood as well.”*

While the participants often referred to oral health, the visit was dominated by child development milestones identified in the “Red Book.” References to “breastfeeding,” “sleeping,” “generic development,” “abuse,” and “mother's mental health” dominated in the discussions and were identified as a core component of the 9–12-month visit.

Research data suggested that a universal visit usually takes 60–75 min. Within this time, professionals are expected to cover all topics identified in the Personal Child Record. Participants' accounts indicated that this timeframe is usually insufficient for an in-depth discussion of all child development topics. Consequently, some topics are prioritized as needing to receive more attention while others are consequentially marginalized.

“*We struggle all the time. We have to kind of assess what we would focus on with each family. Whether it is breastfeeding, weaning, development or postnatal depression. There are so many things and we don't know what we're walking into when we walk into a house. We could be walking into anything. So we have to kind of assess what we think is the most important thing at the time.”*

It was evident that alongside the Personal Child Record guidance, parents' concerns regarding child development are important and shape the focus of the conversations:

“*When you go to a nine month review they want to talk about feeding, they want to talk about sleep. You know, there's often lots of things. Once you've discussed all those things and then you've got to get to your next visit”*

In such instances, oral health becomes a secondary issue:

“*So, if a parent comes in with a specific problem, it might be about sleep or something, you do devote an awful lot of time to that. And then other things, it's kind of a quick mention. I think that is a real difficulty isn't it”*

The need to cover topics identified in the Personal Child Record alongside those prioritized by parents within a limited time often creates information overload. As a result, it is a difficult to remain focused and maintain the conversation. Whilst the most prioritized topics are addressed at the beginning of the visit, child development aspects that are perceived as less important, including oral health, are touched upon at the end. Consequently, parents' information intake becomes lower and disrupted:

“*They've actually talked about what they wanted to talk about. The teeth aren't that high on their agenda”*

“*You can see them switch off can't you”*

“*Especially if they haven't got any [teeth] yet”*

“*They switch off. I mean you just feel like, yeah, and you just feel like you're just chatting away and nobody's listening and you just, you know”*

From the public health perspective, individuals' behavior change guided by professionals is important for preventing oral diseases. When parents are provided with appropriate health knowledge, they are more likely to be motivated and have relevant skills to maintain good oral health practices (38). However, the discussion suggested that limited time for the home visits positions the Personal Child Record guidelines and parents' preferences as key direction for oral health conversations. This creates information overload and minimizes parents' ability to absorb information needed to build relevant knowledge and skills. Consequently, this limits chances for behavior change and sustaining good oral health practices (see Supplementary Figure 1).

### Finance Cuts and Limited Resources

Research data suggested that recent NHS and local government financial cuts have impacted on the availability of both oral health (OH) resources and human resources in some parts of England. This in turn has shaped professionals' conversations with parents. Regarding OH resources, the participants noted that, unlike in previous years, professionals working in different settings and organizational clusters do not have the same materials for delivering oral health interventions. Some participants stated that they previously had pictorial information to facilitate the discussions, as well as leaflets for parents to refer back to information when needed. This was not the case in current practice where some participants reported having a toothbrush and toothpaste for distribution, whilst other participants reported having no available OH resources.

“*Nothing for oral health. We don't sort of have anything like that for them to keep or to refer back to. We give them so much information all in one go and then we walk out…”*

Another participant echoed the practice and emphasized the importance of providing parents with not only “tools” (toothpaste and toothbrush) for looking after their child's teeth, but also resources leading to a better understanding of the behavior:

“*You know, it's not just a case of giving out a toothbrush and saying ‘brush your teeth'. You know, [there are] little resources to say why it is important”*

Limited or absent OH resources prevent health visitors from enabling parents to better understand the importance of toothbrushing and healthy eating.

“*I used to find those really useful because they were pictorial and, you know, parents could see just what we were trying to tell them.”*

As a result of the recent cuts in finance, several NHS organizations in England have transitioned from paper-based material to electronic resources. The participants reported that instead of carrying demonstration kits and leaving leaflets, they now must download the resources to their laptops or smartphones and show these to parents. This means that while during the visit health care professionals have some resources to facilitate the discussions, the only resources they can refer a family to are online. Participants pointed to the importance of leaving printed materials for parents to read after the home visit. Numerous reasons were described, including some children with no erupted first teeth at the 9–12 months home visit and thus oral health was not perceived as currently relevant; parents' more pressing priorities; and some families' limited access to technologies and the Internet. Furthermore, some participants noted that while previously they were able to print out and thus provide materials for parents who were in digital poverty, due to the limited access to printing facilities in their organizations, they were unable to provide any handouts, including to their most vulnerable client groups.

Some of the participants reported that they did not have access to either paper-based or online resources:

“*So iHV's [Institute of Health Visitor] a website you can go on and you have to pay to subscribe. They do leaflets for parents and leaflets for us. So we'd be able to advise parents if we weren't up on knowledge about something. So we had access to this for a year and then the funding got rejected.”*

Although the participants were positive about resource kits available for group training for health visitors (e.g., over-sized demonstration toothbrush and mouth, demonstration of rotten teeth, free-flow cups, etc.), they noted that they were not suitable for individual family conversations, as they were too big and oriented to group teaching. Furthermore, the procedure of borrowing the resource kit was identified as another factor impeding their effective use in the 9–12-month reviews. Specifically, the kit must be pre-ordered in advance, collected from storage, transported to a family's house and then returned to the responsible site. The participants' accounts suggested that while some parts of the kit might be useful for providing advice to families that have more oral health concerns, it was rarely used due to intense workload and limited time.

A recent staff reductions seemed to contribute to a limited focus on oral health at the 9–12 month visits. Participants reported that organizational efficiencies had led to change in how services were delivered. Specifically, because of staff shortages some visits that used to be carried out in families' homes are now conducted via phone or in clinics. At sites where the number of health visitors is low, only families that face challenges, such as child neglect or safeguarding receive home visits. At these visits, the focus is predominantly on key concerns and well-being, leaving oral health aside. Other health visitors noted that they see families of children aged 9–12 months in local clinics. Even though time for traveling to a family's house is saved, staff availability is still insufficient to provide comprehensive and high-quality advice that also incorporates toothbrushing and healthy eating guidance.

“*When they do see us in clinic you've always got that opportunity [to talk about oral health] but as we were talking before, if we're gonna get twenty people and you've got one health visitor coming to a clinic then how on earth are you gonna do that. […] That's the difficulty and we are unfortunately now in a more difficult situation than we were years ago because we don't have as many staff”*

It was suggested that, in some areas, decreased funding to local authority public health budgets had a direct impact on availability of both the OH resources and health visitors. The synergy of the two factors limits opportunities to provide parents with knowledge and support needed for adopting appropriate oral health practices.

### Oral Health Knowledge and Skills

All health visitors demonstrated some gaps in their oral health knowledge. It was evident that while most of the participants had a good underlying knowledge about toothbrushing and healthy eating, some of them were unsure how to apply this in practice.

“*When my clients say to me ‘a pea size amount', what size pea do I tell them? Is it a marrow fat pea or is it a petit-pois?”*

Another participant knew that evening toothbrushing should be conducted after the last meal/feeding, but was not sure what the recommended time space in between the two is:

“*So what would be the best advice because somebody asked me this? When they have the night time bottles they want to brush and then give a bottle. So I said no you should brush after the bottle but then you shouldn't be brushing straight after the bottle should you?”*

Participants' accounts suggested an awareness of parents' struggles in managing their child's behavior while brushing, as well as uncertainty about what advice to provide. Most of the participants were aware of aids, such as mobile apps, songs and TV programmes that may assist parents/carers in managing behavior. However, some of the health visitors reported uncertainty in advising parents how to manage their child's behavior:

“*Going back from what you said about how to brush their teeth. I know some parents will hold their baby down because they don't like it. And then they get issues with food and things around their mouth, phobias. So how would you, if they didn't like the toothbrush and brushing, if they're gonna do it for two minutes, how would you expect, recommend to a parent on how to get them used to it?”*

Education is important for successfully implementing knowledge into practice and professional development (52, 53). It can be argued that the current limited practice of providing health visitors with oral health training and education contributes to their misconceptions of toothbrushing and oral health. For instance, many participants noted that oral health was not a part of the curriculum when training to become a health visitor.

“*I can't remember in my course getting particular training on dental hygiene”*

While training on oral health is a part of health visitors' professional development, it seems to be a one-off and often a self-led research-oriented practice.

“*We have to do a certain amount of training days or research into different areas of our job role. From my memory I think I attended a dental course to gather my research and information. But I haven't done anything since, I haven't been on one since”*

It was evident that training provision is neither systematic nor regular. Instead of being centrally organized and uniformly delivered to all professionals, it seems in some areas to be more coincidental than strategically planned.

“*It's not a mandatory training. It's not something that we have to do every year or every two years. And it's just complete coincidence, somebody rang the other day when I was on, covering duty and said, “can I come and deliver a training session [on oral health] to the health visitors”. So I've just invited her into our monthly meeting.”*

The participants often described oral health training as being “in the background” and it was evident that its initiation and organization usually depends on health visitors' own initiative and capacity. Some organizational clusters have a health visitor whose role involves gathering available research and communicating it to the other team members. Others noted that they occasionally dedicate part of the team meetings to share knowledge gathered either in training or everyday professional practice. Similar practices seem to be present regarding learning how to use available resources used in home visits:

“*We've just got to, like, we've got to muddle through and find a way”*

Regarding learning how to use electronic resources, the participants reported absence of available training and support. Consequently, they usually seek for assistance from more technologically literate colleagues or employ personal contacts, such as friends or family members:

“*It's alright if you have a young person in your team, and we did have one until a few years ago. She would just set us all up, and show us all. […] But she just left, traveling. So, you know, you have to find an asset like that, and to find something like that that can work for you. So find us a new computer literate”*

“*So, what'll happen is one of us will have to get our child to do it, and bring it in, you know”*

Participants' narratives suggested that neither educational institutions that train health visitors, nor organizations that employ the professionals in some areas in England provide systematic and comprehensive training on oral health. Likewise, the responsibility of gaining knowledge about how to use available resources is left to individual health visitors or the local teams they work in.

### Collaborative Working With Other Professionals

The participants identified collaborative work with other professionals as a potential partnership through which to provide parents of young children with oral health advice and support in adopting appropriate home-based oral health behaviors. Discussions focused on the challenges and opportunities of working together with children's centers, general practitioners and dental practices. In relation to children centers, it was evident that some collaborative work is already present. After recent merges of small health visiting teams into larger clusters many baby clinics have been moved to children's centers and this is a place where members of health visiting teams often conduct developmental reviews. The participants viewed working with other professionals in children's centers as enabling further collaboration. They also noted that consistent knowledge among the professionals is important in order to ensure quality of such work.

“*I think that we could have something a little bit more permanent within the children's centers, with the children center staff and the health visiting wider team. And I think it'd be really useful for children's center staff if there was any training, all of us access the same training.”*

Other participants echoed such a position and noted that this practice would minimize their workload and enable focusing on developmental issues that are perceived as priorities. It would provide parents with an opportunity to receive oral health advice that is less restricted than a one-off visit by a health visitor.

“*I think possibly the children centers would probably have a better workforce or capacity to follow up stuff like that [provision of oral health advice] than what we have. I mean yeah possibly the nursery nurses, it might be something for them to do but everyone is struggling with the workload that we've got.”*

In a similar vein, a number of participants noted that since some parents see general practitioners (GPs) regularly, provision of oral health advice in this health care setting may contribute to raising parents' awareness of appropriate oral health behaviors. However, some participants reported that GPs often lack relevant knowledge and may provide parents with misleading advice.

**Table:** 

*Researcher:*	*“Do you have practices of putting condensed milk, sugar or juice [in bottles]?”*
*Participant:*	*“Not so much. Juice yes, but not so much the condensed milk. Sugar…I think we get the sugar when they're teething. Possibly not as much as we used to. I think we are sort of starting to steer away from the sugar in bottles. I have had a couple of GP's that have suggested it for constipation which has driven me insane! So yeah so obviously we still have to educate the GP's as well”*

While the focus in the participants' narratives was on professionals working in children's centers and GPs' sometimes limited knowledge on oral health, the accounts suggested openness and potential for interdisciplinary collaboration. On the other hand, their experiences with, and position regarding collaboration with dental practices was at the opposite end of the spectrum. While local governments are increasingly calling for more collaborative work between dental services and health visitors (54), many of the participants noted that their engagement with local dental practices is limited. They positioned the lack of cooperation as one of the barriers for engaging with parents in oral health conversations and encouraging toothbrushing. In particular, the participants noted difficulties related to the lack of NHS dentists in the area, meaning that parents often struggle to register their young children with dentists and/or to get appointments and have their teeth examined. This further prevents parents' oral health education and may lead to withdrawal from seeking the service:

“*We've got a real problem around here, there's just not enough provision. They [parents] need to be really tenacious if they're gonna get a dentist. And some of them just don't have the enthusiasm to keep trying. Cause we've only got really two dentists, haven't we, that'll take on NHS patients that's, that are local?”*

Participants' accounts suggested that children of parents who do access NHS dentists often do not receive any dental advice or care until they are older. Even if parents do take their children to a dental practice, they often do not have the opportunity to familiarize with the setting and procedure, or to have their teeth checked.

“*They get the things about taking, if it's, they're having an appointment they'll say, “oh I'll take them along so they get used to it”. And then I say, “well did the dentist put them in the chair just to get used to the chair? Did they attempt to ask them to open their mouth? Or did they get them to put the sunglasses on?” And a lot of time they'll say “no, they just came along and said hello to them”. So don't get me wrong, not all, you know”*

This was a common practice and the participants often reported it being frustrating and contradicting their advice to seek regular dental examinations and ongoing care. Specifically, while the participants reported encouraging parents to register with and take their young children to a dentist, limited provision of the service decreases the potential impact of the advice provided. Participants' accounts suggested that in order to achieve a common goal of better oral health in young children, dental professionals should take part of the responsibility and play a more active role. However, it was evident that health visitors' attempts to collaborate, directly or indirectly, with dentists often are unsuccessful.

## Discussion

Our study is an important step in understanding how organizational factors within current public health policy and service provision shape oral health conversations between health visitors and parents of young children aged 9–12 months old. Traditionally, research has focused on the cognitions and behavior of individuals, but every individual operates within a complex social and physical environment. Moreover, we are influenced and, in some instances, constrained by various community and organizational environments. This study highlights some of the public policy mechanisms and processes that are experienced by health visitors and how this in turn impacts on parents of young children.

Conventionally, preventative dentistry has focused on attempting to change the oral health behaviors of high-risk individuals, but such a narrow focus has failed to remedy the gap in oral health inequalities (55). The problem lies in that, although individual oral health conversations, as well as training the wider workforce is useful, it is a “downstream” approach, which is ultimately influenced by the organizational and contextual barriers at a more “upstream” level (55). This study clearly demonstrates that, without adequate “upstream” support, the impact of the “downstream” oral health conversations are tempered. Therefore, as stated by Watt [(55): 5] “the central focus for action needs to be the creation of a social environment which facilitates and maintains good oral health across the population.”

Recognizing the interaction between intervention content and the context in which it is implemented is becoming increasingly important in order to effectively transfer interventions to different contexts (56). Indeed, any complex intervention will likely need to adapt to the context in which it is implemented and understanding the wider context and system may aid the development of facilitation strategies (57). Subsequently, this is why the generic intervention we developed (42), from which our subsequent HABIT (Health Visitors delivering Advice in Britain on Infant Toothbrushing) intervention (58) is derived, was mapped out considering each socio-ecological level (individual, interpersonal, community, organizational, and environmental). The current study contributes to the literature by providing a detailed account of the organizational barriers to the current oral health advice delivered by health visitors to parents. Each of which will now be discussed in turn.

Health visitors acknowledge the importance of child oral health and address it in their universal home visits at 9–12 months. Nevertheless, they focus on the topic significantly less than they do on other aspects of child development. Our findings suggested that the Personal Child Record (“Red Book”), current requirement in the area from which the participants were drawn, provides a framework for universal home visits. This is interesting as being aimed at parents, it also drives health visitors' focus and approach to the conversation. The Personal Child Record only briefly addresses oral health and is focused on factual information rather than guidance and advice. This, in conjunction with the need to cover all child development aspects within a limited time frame, is one of the reasons why oral health conversations between parents and health visitors often lack depth and in some instances are only briefly touched upon, aiming to “tick the box” that the subject has been covered. As such, it is not surprising then that parents' expectations for the 9–12-month developmental visit are similar to the framework provided in the Personal Child Record. In the current study, participants reported that parents are rarely interested in oral health or emphasize it as a priority for the visits. On the other hand, study conducted by Filipponi et al. (59) suggest that parents from a deprived area in Wales were involved in the Government's scheme aimed delivering supervised toothbrushing and oral health education while parents from least deprived area did not took part in the scheme as it was not perceived as important enough or appropriate. Our study, however, involved parents from one of the most deprived areas in England. As discussed earlier, key topics for the universal home visits at 9–12 months usually included child's growth or weight; vision or hearing; sleep routines; behavior; and child development. These indeed are the “Red Book” priorities and parents' needs and preferences related to child development issues that have to be addressed within a limited time frame and are usually discussed at the beginning of the visit. This often creates information overload that limits parents' information intake. This strengthens the portrayal of oral health as not important as parents remember and relate to the information that is provided first rather than last (the primacy effect). A potential avenue to overcome the issue is to use the universal service to signpost families, especially those children who are at higher risk of developing tooth decay, to universal plus services. Such a practice would allow for a collaborative practice and facilitate a creation of a personalized intervention which fits the family's social environment and thus is more likely to lead to appropriate oral health practices and behaviors. Participants' accounts suggested that, in some areas of England, the Local Authority funding gap has influenced the quality of oral health conversations between health visitors and parents of young children aged 9–12 months old. The policy and practice of cost efficiencies was evident and manifested frequently in the participants' narratives. Participants' highlighted that there is limited or absent availability of resources to be used for communicating key oral health messages. Furthermore, considering fluctuating numbers of health visitors it may be suggested that the health visiting service is under-equipped to meet current demand. Fewer health visitors means less opportunity to engage in oral health conversations that enable parents to better understand why, how and when child oral health matters, and how to ensure good oral health behaviors are practiced.

Barriers to good quality oral health conversations are re-inforced by a lack of knowledge and training provision on oral health for health visitors. Neither health visitor training courses nor professional development programmes have a systematic and in-depth focus on oral health. Hence, health visitor educators should devise programmes that cover topics that are perceived as less important both in the pre-professional acquisition phase as well as while working as a health visitor. While limited provision of regular and systematic training on the subject encourages health visitors' collaboration and autonomy, it limits their time for visiting families and providing necessary advice. It is therefore unsurprising that health visitors welcomed the opportunity for interprofessional working, particularly with other health and early years professionals. Sharing the responsibility of oral health provision would not only reduce the burden on health visiting services, but also provide the opportunity to improve the reach and reinforcement of oral health messages. However, as the health visitors in the present study acknowledged, ensuring consistency in knowledge and message delivery amongst all professionals would be of paramount importance.

Upon identifying these barriers, we sought to rectify them through the creation of the HABIT intervention (58). This was achieved through co-production, a process which actively involved health visitors in the design and development of the intervention. Such a process facilitated the development of an intervention that considers both “downstream” and “upstream” factors. Although the focus of this paper and subsequently developed intervention is on improving oral health, the methodology could be transferred to other public health concerns. It is highly likely that the organizational barriers experienced by health visiting teams, namely the lack of training, funding, time and resources, are equally applicable to a range of services. It is hoped, therefore, that this research will provide a base upon which to build and improve future public health research and intervention development.

The main limitation of the present study is that the interviews/focus groups were only undertaken within one area of England, thus potentially limiting the transferability of the results. However, consultation with the Institute of Health Visiting revealed that what we found was an accurate reflection of what is also happening in other services across England.

In conclusion, this is the first paper to explore the organizational barriers influencing the oral health conversations between health visiting teams and parents of young children. The findings highlight that to effectively intervene, both “downstream” and “upstream” approaches must be unified. This must, however, be achieved in a pragmatic and empathetic way that utilizes and modifies existing entities (e.g., training and resources).

## Data Availability Statement

The raw data supporting the conclusions of this article will be made available by the authors, without undue reservation.

## Ethics Statement

The studies involving human participants were reviewed and approved by University of Leeds Dental Research Ethics Committee (DREC: 160517/PD/229). The patients/participants provided their written informed consent to participate in this study.

## Author Contributions

PD is the principal investigator for the grant and led the development and writing of the trial protocol. KG-B, ZM, SP, and RW contributed to the study design, methods and writing of the trial protocol. IE led the ethics application, data collection, analysis and writing of the manuscript with major contributions from PD, KG-B, JO, and ZM. IE, JO, BS-M undertook data collection, data cleaning and monitoring aspects of the protocol. All authors read and contributed to the writing of the paper and have read and approved the final manuscript.

## Conflict of Interest

The authors declare that the research was conducted in the absence of any commercial or financial relationships that could be construed as a potential conflict of interest.
