# Supplementary material for: Organizational Barriers to Oral Health Conversations Between Health Visitors and Parents of Children Aged 9–12 Months Old
Source: Front Public Health. 2021 Feb 23;9:578168. doi: 10.3389/fpubh.2021.578168 (PMC7940188; doi:10.3389/fpubh.2021.578168)

Supplementary Material

1 Supplementary Figure 1 Challenges to behaviour change conversations

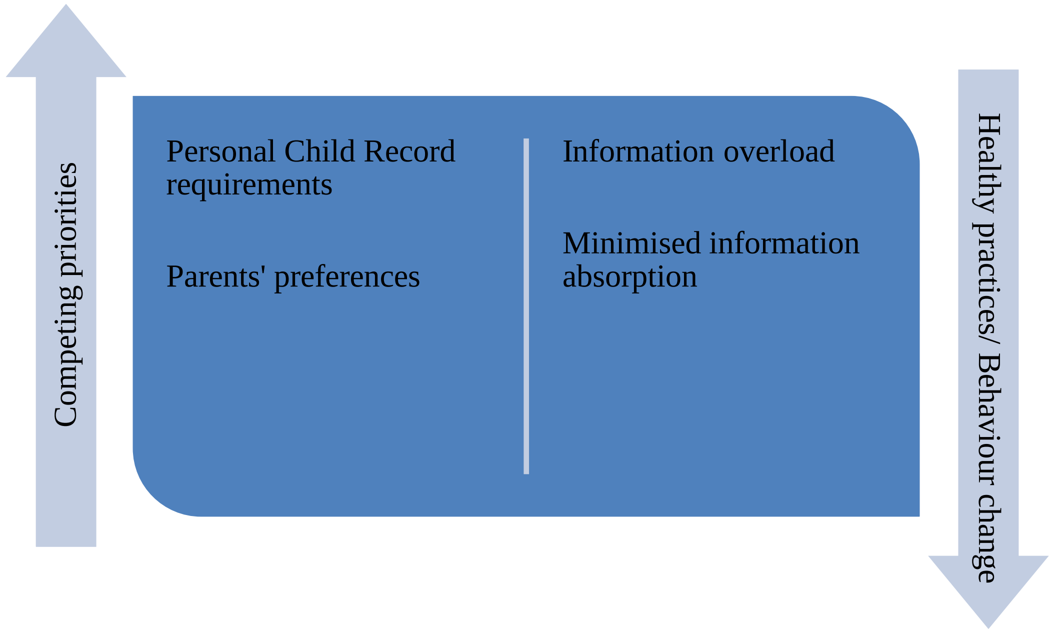

Supplement: Supplementary file 1 [file Data_Sheet_1.PDF]
